# Incidence and Etiology of Surgical Site Infections among Emergency Postoperative Patients in Mbarara Regional Referral Hospital, South Western Uganda

**DOI:** 10.1155/2017/6365172

**Published:** 2017-01-12

**Authors:** Abubaker Lubega, Bazira Joel, Najjuka Justina Lucy

**Affiliations:** ^1^Department of Surgery, Mbarara University of Science and Technology, Mbarara, Uganda; ^2^Department of Microbiology, Mbarara University of Science and Technology, Mbarara, Uganda

## Abstract

*Background.* This prospective hospital based study was conducted to determine the incidence, risk factors, and causative agents of surgical site infection their susceptibility to among 114 emergency postoperative patients at the Mbarara Regional Referral Hospital between September 2014 and January 2015.* Methods*. Consented patients were consecutively enrolled and their preoperative, intraoperative, and postoperative data were collected. Follow-ups were done in the surgical outpatient clinics. Wound specimens were collected and processed as per Sops; susceptibility testing was done using the Kirby-Bauer disc diffusion technique. Data was analyzed using STATA 11.0.* Results*. Overall SSI incidence was 16.4%: 5.9% superficial and 47.1% deep and organ space SSIs each.* Klebsiella pneumoniae* was the most predominant organism (50%) followed by* Staphylococcus aureus* (27.8%).* E. coli* and* P. aeruginosa* both accounted for 11.1%. Wound class (*p* = 0.009), anaemia (*p* = 0.024), low serum albumin (*p* = 0.046), and property of suture material used (*p* = 0.006) were significantly associated with SSIs. All organisms had 100% resistance to ampicillin, tetracycline, septrin, and erythromycin. Ciprofloxacin and ceftriaxone are highly sensitive to all organisms.* Conclusion*. The incidence of SSI in this hospital is very high.* Klebsiella pneumoniae* is the predominant cause. Ciprofloxacin are very potent antibiotics against organisms that cause SSI.

## 1. Introduction

Surgical site infection (SSI) is an infection that develops within 30 days after an operation or within one year if an implant was placed, and the infection appears to be related to the surgery [1].

SSIs remain a major cause of morbidity and death among the operated patients and continue to represent about a fifth of all healthcare-associated infections [1]. Although at least 5% of patients develop an SSI after surgery [2], these infections seem to cause remarkably little concern, remaining largely unreported in the media.

Despite improvements in operating room practices, instrument sterilization methods, better surgical technique, and the best efforts of infection prevention strategies, surgical site infections remain a major cause of hospital-acquired infections and rates are increasing globally even in hospitals with most modern facilities and standard protocols of preoperative preparation and antibiotic prophylaxis. Moreover, in developing countries where resources are limited, even basic life-saving operations, such as appendectomies and cesarean sections, are associated with high infection rates and mortality [3].

In the developed countries, SSI has been reported to affect from 5% to 15% of hospitalized patients in regular wards and as many as 50% or more of patients in intensive care units (ICUs), while in developing countries the magnitude of the problem remains largely underestimated [4].

In Uganda, data about SSI is still scarce and the true incidence and cost per patient are unknown. In MRRH, research done to determine the incidence of SSI among elective surgeries on the surgical ward in 2007 found the postoperative incidence density to be 15.9% and no risk factors were associated with SSIs.

Patients who develop SSI require significantly more medical care. If an SSI occurs, a patient is 60 percent more likely to spend time in the ICU after surgery than is an uninfected surgical patient and the development of a SSI increases the hospital length of stay by a median of two weeks [1].

The risk of SSIs continues after discharge. SSIs develop in almost 2 percent of patients after discharge from the hospital and these patients are two to five times as likely to be readmitted to the hospital [1].

In 2012, major emergency operations contributed more than 43% of the total surgical operations in MRRH (theater records, 2012). Most of these patients are at risk of getting SSIs postoperatively because of the nature of their disease pre operatively, surgical aseptic technique, and underlying comorbidities among others.

Previous studies in this department have tried to shed light on cause of SSI among elective surgical postoperative patients while others have identified the causative organisms of small bowel perforation, a common diagnosis among patients who undergo emergency operations in this department; however, no research has been done to determine the incidence of SSI among emergency postoperative patients. To remedy this lack of data, this study used standardized documentation to provide data of the microbiological etiology, incidence, and risk factors associated with SSI among patients who underwent emergency operations in MRRH and would be used to establish the sensitivity patterns of organisms to commonly available antibiotics.

## 2. Methodology

### 2.1. Study Site

The study was conducted in Mbarara Regional Referral Hospital's surgical department, a 350-bed capacity government funded hospital located 260 km along Kampala-Kabale high way with a staffing of about 410 persons.

### 2.2. Study Design and Duration

It is a prospective cohort study in which participants who underwent emergency surgical operations were followed up for a maximum period of 30 days postoperatively between September 2014 and January 2015 at Mbarara Regional Referral Hospital.

### 2.3. Study Population

Study population was all patients admitted to Mbarara Regional Referral Hospital and underwent emergency surgery and those who had previous emergency surgery at MRRH and were readmitted with wound sepsis within 30 days after operation.

### 2.4. Sample Size

The sample size was calculated using open epi software (The Fleiss formula by John Wiley & Sons, 1981) with level of significance (*α*) of 5% (0.05) and power of the study (1-*β*) of 80%. Total sample size for this study was 114 patients.

### 2.5. Study Procedures

Following resuscitation and stabilization, admitted patients who met the inclusion criteria were recruited into the study. Written consent to participate in the study was then obtained and a full history and physical examination done. Patient data was collected using a pretested interviewer administered semistructured questionnaire.

Data collected included patient sociodemographics, past medical history, previous treatment, the time from presentation to time of operation, cadre of operating surgeon, and duration of surgery.

### 2.6. Laboratory Procedures

In patients who developed SSI, exudates from wounds were collected using sterile swabs which were transported at room temperatures to the laboratory within 15 minute of collection. Swabs were inoculated on chocolate, blood, and MacConkey agar. Chocolate plate was placed in a candle jar and incubated with other plates at 35–37°C for 24–48 hours. A Gram stain procedure was performed on culture growths to report the organisms.

An additional blood agar plate was inoculated anaerobically at 35–37°C for 48–72 hours. Plates were examined aerobically for potential pathogens like* Staphylococcus aureus, beta Haemolytic streptococci, Enterobacteriaceae, Pseudomonas aeruginosa, Acinetobacter species, Haemophilus influenzae, *and* Streptococcus pneumonia.*

Susceptibility testing was done by the Kirby-Bauer disk-diffusion method. Methicillin resistance among staphylococcal organisms and extended spectrum producers among Gram-negatives were not tested because they were beyond the scope of this study.

### 2.7. Statistical Analysis

Data was entered to epi info 7.0 and checked for errors and analysed using STATA 11. Analyzed data was presented in tables and figures showing frequencies and proportions.

Univariate analysis was done for continuous variables to report measures of central tendency like mean and median, measures of dispersion like the range and interquartile range, and measures of variance like standard deviation for various independent variables. For categorical data, bar graphs and pie charts were used to present the data.

Bivariate logistic regression as well as multivariate logistic analyses and Pearson chi-square *χ* ^2^ were be applied to determine associations. The level of significance was preset at 5%.

Odds ratios (ORs) with their respective 95% confidence intervals were used to assess for statistical associations and *p* values of less than 0.05 were considered statistically significant.

### 2.8. Ethical Approval

The study was approved by the department of surgery of Mbarara University of Science and Technology, Faculty Research Committee and the Institutional Review Committee on Mbarara University of Science and Technology. All participants were provided with a written informed consent.

## 3. Results

### 3.1. Study Participant Social Demographic Characteristics

Overall a total of 114 patients were recruited and followed up for 30 days from September 2014 to January 2015 for the study. Four (4) of these patients were lost to follow-up leaving 110 patients for analysis. Of these, 82 (74.5%) were males and 28 (25.5%) were females.

Table 1 shows that most patients came from Mbarara, were peasants, and had attained only primary education and were either single or cohabiting. The median age of the cohort was 26 years with an IQR of 29 years and range of 2 months to 88 years.

### 3.2. Incidence of SSI

The incidence of surgical site infection was 16.4% with a 95% confidence interval of 9. 3 to 23.4%. Eleven (11) (3.4%) of the 82 males and 7 (25%) of the 28 females got SSI.

Superficial SSI accounted for 5.9%, whereas deep and organ space SSI accounted for 47.1% each.

Patients undergoing operations for small bowel perforation, gastric perforation, and intussusception had the most SSI whereas those for ruptured spleen, sigmoid volvulus, and acute epidural hematoma had no SSI.

### 3.3. Etiology of SSIs

Four organisms were responsible for causation of SSIs among this cohort.* Klebsiella pneumoniae* caused 50% of the SSIs followed by* Staphylococcus aureus* at 27.8% and* E. coli* and* Pseudomonas *shared 11.1% each.

### 3.4. Risk Factors Associated with SSI

At bivariate logistic regression, low serum albumin (*p* = 0.004), Hb levels < 8 g/dL (*p* = 0.008), use of drain (*p* = 0.036), dirty wounds (*p* = 0.004), using chlorhexidine as skin scrub (*p* = 0.032), using absorbable suture material (*p* = 0.039), and the ASA score (*p* = 0.022) were associated with SSIs. However, giving antibiotics prior to admission, use of preoperative antibiotics, and all demographic factors were not associated with SSI (Table 2).

After multivariate logistic regression, skin antiseptic used to scrub the surgical site, use of drain, property of suture, and ASA score were found to be insignificant and were dropped. The final model consisted hence of serum albumin (*p* = 0.046), hemoglobin level (*p* = 0.024), type suture material used (*p* = 0.006), and CDC wound classification (*p* = 0.009) as shown in Table 3.

### 3.5. Sensitivity Patterns of the Causative Organisms

In total, 10 drugs were tested for sensitivity to the identified organisms. 40% of these drugs had 100% resistance to the organisms; these included ampicillin, tetracycline, septrin, and erythromycin. The remaining 60% drugs had varying sensitivity patterns.

Gram-negative organisms were sensitive to ciprofloxacin and ceftriaxone and had varying resistance for gentamycin and Nalidixic acid whereas they were highly resistant to chloramphenicol. Of the Gram-negatives,* E. coli* had the highest resistance to all antibiotics.

*Staphylococcus aureus* was the only Gram-positive isolate identified. It had 100% sensitivity to both ceftriaxone and gentamycin but was highly resistant to chloramphenicol.

Ciprofloxacin and ceftriaxone had the best sensitivity with *p* values of 0.048 and 0.009, respectively. Chloramphenicol had the worst resistance to both Gram-positive and Gram-negative organisms (Table 4).

## 4. Discussion

Postoperative SSI remains one of the most important causes of morbidity in surgically treated patients. These patients incur higher cost because of longer hospitalizations, more nursing care, additional wound care, potential readmission to the hospital, and further surgical procedures.

As in most studies in Africa and other developing continents, the incidence of SSI in our study was high at 16.4%. This contrasts with the overall SSI rate in mainland China which is at 4.5% [5] and that in Seoul, South Korea, at 3.3% [6] and that in the US at 2-3% [7]. In Ethiopia, SSI rates were found at 10.6% [8] whereas in a private hospital in Kenya incident rates of 7.0% were found [3] and the overall SSI rate in Nepal and Tanzania was 23% and 26%, respectively [2, 9].

These findings reflect a lack of adequate postoperative care and failure to maintain sterility during surgical procedures, inadequate infection control due to poor hygiene, resource and structural constraints, and lack of awareness regarding nosocomial infections among the general population.

Technological advances in infection control like use of high-efficiency particulate air (HEPA) filters in theaters to reduce bacterial loads are still lacking in the African setting and this in part may contribute to the high incidence of SSI.

Therefore, the high standards of health care in the developed countries remain as the most credible explanation to this difference in the rates of infection observed.

In this research, wound class, anaemia, low serum albumin, and property of suture material used were significantly associated with surgical site infection.

Surgical wound classification has long been established as an important predictor of the postoperative surgical site infections [3, 7, 10]. In our study, the risk of SSI was statistically higher in dirty and contaminated wounds than in clean and clean contaminated wounds. Our findings confirmed previous knowledge that surgeries with an increased microbial load in the operative field are associated with higher risk of SSI.

The property of suture material used on skin was significantly associated with SSI. The risk of SSI was high when an absorbable suture was used than when a nonabsorbable suture was used. The risk ratio was also true when a braided was used. These findings are concordant with those in Nepal [2].

The odds of developing SSI were higher among patients with low serum albumin compared to those with normal levels. The result is consistent with findings in Sweden [11]. Serum albumin is an indicator of the patient's nutritional status. Malnutrition is a well-documented risk factor for SSI. Malnourished patients are at risk of impaired systemic and intestinal immune function, as well as decreased digestive and absorptive capacity due to the altered architecture of the gut barrier.

A deficiency of protein can impair capillary formation, fibroblast proliferation, proteoglycan synthesis, collagen synthesis, and wound remodeling. A deficiency of protein also affects the immune system, with resultant decreased leukocyte phagocytosis and increased susceptibility to infection.

As documented in previous studies, the level of hemoglobin concentration was significantly associated with SSI. Severely anemic patients had the highest risk of getting SSI.

This is consistent with findings of in Nepal [2]. A low hemoglobin concentration creates the risk of SSI through tissue hypoxia and impairment to tissue healing.

In this study, the odds of developing SSI when using chlorhexidine were high compared to using iodine at univariate logistic regression but insignificant at multivariate regression. In contrast, the study in Tanzania found iodine alone to be associated to SSI than when used in combination with either chlorhexidine or alcohol related solutions [9].

Smoking has been shown to be an independent risk factor for SSI [2, 4]. Smoking delays the healing of SSIs by causing local and systemic vasoconstriction. This results in tissue hypoxia and hypovolemia, an environment conducive to SSI.

Heavy alcohol consumption weakens immunity and increases the risk of SSI, although this effect is dose-dependent and was not addressed in our study. The above risk factors were not significant associated with SSI in our study.

Duration of operation time was not significantly associated with SSI; however, previous studies have also assessed the influence of prolonged operative time as a risk factor for SSI [2, 9, 12].

A prolonged operative time leads to fatigue, resulting in a decline in the use of aseptic measures during surgery and may also be associated with advanced disease, reoperation, or intraoperative difficulties. Additionally, a prolonged operative time is often related to increased blood loss which contributes to tissue hypoxia.

In contrast to other studies, the predominant cause of SSI in our study was* Klebsiella pneumoniae*; causing most of the SSIs this was followed by* Staphylococcus aureus, E. coli,* and* Pseudomonas *spp. caused the least SSI. Several studies in Ethiopia, Gabon, Cameroon, Nigeria, Tanzania, Kenya, and Mulago National referral Hospital Uganda are concordant with findings of this study [3, 8, 9, 13–16].

*Klebsiella pneumoniae's* natural habitat is the gastrointestinal tract. Most of the operations performed were laparotomies and most wounds were either clean contaminated, contaminated, or dirty; this spillage from the GIT explains why this organism caused the most SSI.

Most SSI were caused by multi-drug resistant (MDR) organisms. Resistance to antibiotics ranged from 11.1% to 100%. This consistent with research in Mulago and Muhimbili, Tanzania [16–18].

*Klebsiella pneumoniae *showed very high sensitivity to both ciprofloxacin and ceftriaxone and Nalidixic acid but was resistant to septrin, ampicillin, tetracycline, and erythromycin.

*Klebsiella*'*s* resistance to septrin was also reported in Tanzania [1]. Resistance to chloramphenicol was also reported in Jinja, Uganda [19].

*Staphylococcus aureus* in this research showed high sensitivities to ciprofloxacin, ceftriaxone, and gentamycin but was resistant to erythromycin, chloramphenicol, and ampicillin. These findings were consistent to those found in Jinja RRH [19].

For ceftriaxone to have such very high sensitivities against both Gram-negative and Gram-positive organisms is surprising since the drug is prescribed to almost everyone who undergoes an emergency operation.

## 5. Conclusion

The incidence of SSI among emergency postoperative patients at Mbarara Regional Referral Hospital is high compared to that in the developed world.

*Klebsiella pneumoniae* and* Staphylococcus aureus* cause the majority of SSI among emergency postoperative patients. Development of SSI was associated with low serum albumin, anaemia, type of suture used, and dirty wound class.

Ceftriaxone and ciprofloxacin are very potent antibiotics against organisms that cause SSI among emergency postoperative patients at Mbarara Regional Referral Hospital.

A surveillance system for SSI with feedback of appropriate data to surgeons and hospital authorities is highly recommended to reduce the SSI rate in MRRH and nutritional support should be given to patients at a high risk of developing SSI.

## Figures and Tables

**Table 1:** Demographic characteristics of study population.

Characteristic		*n* (%)
Gender	Male	82 (74.5)
	Female	28 (25.5)

Age category	<1 year	4 (3.6)
	1–14 years	4 (3.6)
	14–45 years	44 (40.0)
	45–60 years	47 (42.7)
	>60 years	11 (10.0)

Address	Mbarara	38 (34.6)
	Isingiro	28 (25.4)
	Sheema	10 (9.1)
	Others	34 (30.1)

Occupation	Preschool	9 (8.2)
	Student	32 (29.1)
	Peasant	46 (41.8)
	Business person	17 (15.6)
	Others	6 (5.4)

Marital status	Single/cohabiting	57 (51.8)
	Married	48 (43.6)
	Widowed	2 (1.8)
	Divorced	3 (2.7)

Education level	Preschool	23 (20.9)
	Primary	67 (60.9)
	Secondary	14 (12.7)
	Tertiary	6 (5.5)

**Table 2:** Bivariate analysis of risk factors for development of SSIs.

Characteristic	COR (95% CI)	*p* value
Smoking		*0.438*
Yes	0.4 (0.52–3.58)	
Alcohol		*0.805*
Yes	1.1 (0.37–3.58)	
HIV status		*0.337*
Reactive	0.3 (0.43–2.92)	
ASA score		***0.022***
Moderate (2-3)	1.4 (0.47–4.36)	
Severe (>3)	11 (1.51–79.83)	
Hemoglobin levels		***0.008***
Mild anaemia	1	
Moderate anaemia	3.2 (1.05–9.90)	
Severe anaemia	6.9 (1.28–37.66)	
Serum albumin		***0.004***
Normal	6.8 (1.28–25.10)	
Duration of operation		0.249
<1 hr	1	
1-2 hrs.	0.9 (0.99–9.16)	
>2 hrs	1.8 (0.21−16.92)	
Suture material		**0.039**
Monofilament	1	
Braided	5.9 (1.09–32.19)	
Cadre of operating surgeon		0.163
Surgeon	1	
SHO	2.2 (0.725–6.71)	
Use of a drain		**0.036**
Yes	5.1 (1.11–23.713)	
Wound classification		**0.004**
Clean	1	
Clean contaminated	8.5 (0.87–81.65)	
Contaminated	7.5 (0.82–68.25)	
Dirty	19.2 (2.2–167.21)	
Skin antiseptic		**0.032**
Iodine	1	
Chlorhexidine	9.5 (1.21–74.70)	
Property of suture		**0.039**
Non absorbable	1	
Absorbable	5.1 (1.09–32.19)	

**Table 3:** Multivariate logistic regression analysis of risk factors for development of SSIs.

Characteristic	AOR (95% CI)	*p* value
ASA score	1.79 (0.63–5.07)	0.273
Hemoglobin levels	2.4 (1.12–5.34)	**0.024**
Property of suture	2.4 (0.89–24.78)	0.997
Serum albumin	4.3 (1.02–17.84)	**0.046**
Suture material	30.7 (2.7–351.31)	**0.006**
Skin anti septic	9.0 (0.95–85.48)	0.055
Wound classification	2.59 (1.27–5.27)	**0.009**
Use of a drain	2.2 (0.39–12.7)	0.336

**Table 4:** Sensitivity patterns of the different organisms to the different antibiotics.

Sensitivity pattern	*Staphylococcus *	*Klebsiella* spp.	*E. coli*	*Pseudomonas *	Total (sensitivity) (*n*)	*p* value
Ciprofloxacin						
Sensitive *n* (%)	3 (75.0)	8 (88.9)	0 (0.0)	2 (100)	13	**0.048**
Resistant *n* (%)	1 (25.0)	1 (11.1)	2 (100)	0 (0.0)	4	

Ceftriaxone						
Sensitive *n* (%)	5 (100)	8 (88.9)	0 (0.0)	2 (100)	15	**0.009**
Resistant *n* (%)	0 (0.0)	1 (11.1)	2 (100)	0 (0.0)	3	

Gentamycin						
Sensitive *n* (%)	3 (100)	6 (66.7)	1 (50.0)	0 (0.0)	10	**0.29**
Resistant *n* (%)	0 (0.0)	3 (33.3)	1 (50.0)	1 (100)	5	

Chloramphenicol (CAF)						
Sensitive *n* (%)	1 (20.0)	4 (66.7)	0 (0.0)	0 (0.0)	5	**0.194**
Resistant *n* (%)	4 (80.0)	2 (33.3)	2 (100)	1 (100)	9	

Nalidixic Acid						
Sensitive *n* (%)	—	1 (100)	0 (0.0)	—	1	**0.157**
Resistant *n* (%)	—	0 (0.0)	1 (100)	—	1	

Cloxacillin						
Sensitive *n* (%)	4 (80.0)	—	—	—	4	
Resistant *n* (%)	1 (20.0)	—	—	—	1	
